# Anatomical relationship between the intertrochanteric line and femoral neck osteotomy level in direct anterior approach total hip arthroplasty: a 3D morphometric and cadaveric validation study

**DOI:** 10.1186/s42836-025-00365-4

**Published:** 2026-03-03

**Authors:** Sakkadech Limmahakhun, Suchate Runraksar, Nitchanant Kitcharanant, Warakorn Jingjit

**Affiliations:** Department of Orthopaedic Surgery, Faculty of Medicine, Chiang Mai University, Chiang Mai, 50200 Thailand

**Keywords:** Direct anterior approach, Intertrochanteric line, Femoral neck osteotomy, Bony reference

## Abstract

**Aims:**

Inaccurate femoral neck osteotomy is a recognized technical challenge in direct anterior approach total hip arthroplasty (DAA-THA), largely due to limited femoral exposure and the absence of a standardized intraoperative landmark. This study aimed to investigate whether the ITL is an alternative bony landmark for femoral neck osteotomy during the DAA.

**Patients and methods:**

Three anatomical references, the Intertrochanteric line (ITL) height (ITL-H), ITL angle (ITL-A), and femoral saddle height (SH), were measured from 3D-CT models of 60 normal hip patients (30 males and 30 females) to simulate a cutting height of 10 mm above the LT. Twenty cadaveric hip specimens were then used to evaluate the accuracy of the proposed anatomical references.

**Results:**

The mean ITL-H, ITL-A, and SH were 23 ± 4 mm, 17.4° ± 3.5°, and 26 ± 4 mm, respectively. While ITL-H showed no sex difference (23 ± 3.4 mm vs 23.1 ± 4.1 mm, *P* = 0.96), significant differences existed for ITL-A (15.8° ± 3.4° vs 19.9° ± 1.4°, *P* = 0.001) and SH (27.2 ± 3.9 mm vs 23.9 ± 3 mm, *P* = 0.002). ITL-H was not correlated with age (*P* = 0.063), femoral length (*P* = 0.31), or femoral neck shaft angle (*P* = 0.41). Femoral neck osteotomy performed 23 mm above the ITL-H could yield 80% and 100% success rates for cutting heights of 10–15 mm and > 5 mm above the LT, respectively.

**Conclusions:**

ITL-H serves as a reproducible anatomical landmark for femoral neck osteotomy during DAA-THA. An osteotomy level of approximately 23 mm above the ITL-H represents a safe lower margin to avoid excessive calcar bone resection. Nevertheless, individualized preliminary osteotomy based on preoperative templating remains necessary, with intraoperative adjustment according to patient-specific ITL-H.

Video Abstract

**Supplementary Information:**

The online version contains supplementary material available at 10.1186/s42836-025-00365-4.

## Introduction

Total hip arthroplasty (THA) has undergone significant advancements in surgical approaches, with the direct anterior approach (DAA) emerging as a transformative technique for primary hip replacement. Strong evidence supports its benefits, including faster recovery, reduced postoperative pain, and lower opioid consumption, compared with traditional approaches such as posterior or lateral methods [1–3]. These advantages are attributed to the muscle-sparing nature of the DAA, which uses a true internervous plane between the sartorius (femoral nerve) and the tensor fasciae latae (superior gluteal nerve), minimizing soft tissue trauma [4].

One of the key advantages of DAA performed without a traction table in the supine position is the ability to conduct simultaneous bilateral hip replacements and accurately assess leg length and hip length discrepancy through manual measurement and intraoperative fluoroscopy [2, 3, 5]. Despite its benefits, the DAA presents challenges, particularly during the learning curve, which is associated with longer operative times and higher complication rates, including those associated with femoral fractures, lateral femoral cutaneous nerve injuries, and improper implant positioning [2, 3]. Additionally, the limited exposure of the proximal femur makes femoral preparation more technically demanding than the posterior approach, where landmarks are more readily identifiable [6].

**Fig. 1:**
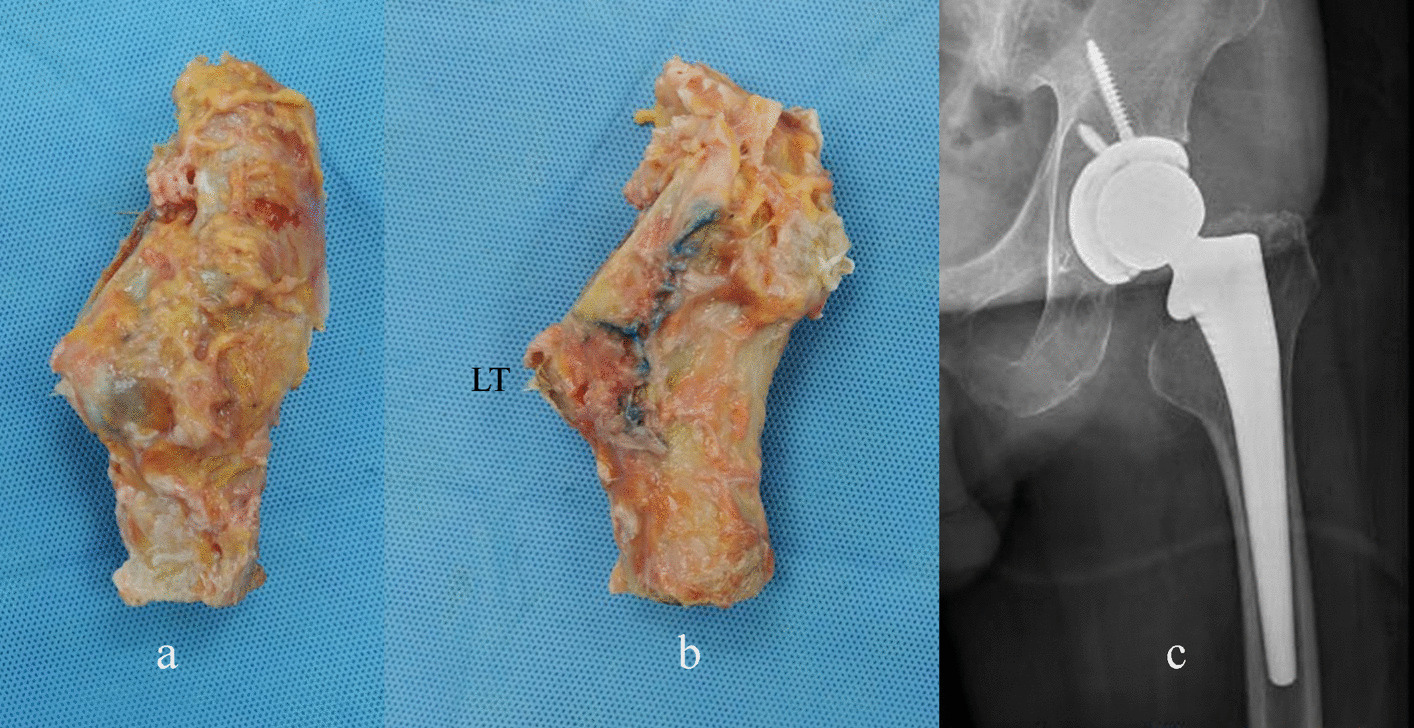
Visualization of the lesser trochanter (LT) from the anterior (**a**) and posterior (**b**) aspects of the proximal femur, demonstrating its relationship to the femoral neck osteotomy (**c**)

Femoral neck osteotomy is a critical step in DAA-THA, which lacks standardized bony landmarks to guide the level and trajectory of the cut. Unlike the posterior approach, where the lesser trochanter (LT) and femoral neck are easily visualized before surgical hip dislocation (Fig. 1), the DAA requires precise identification of anatomical references to ensure proper implant positioning and biomechanical restoration [6].

Recent studies have explored potential bony landmarks for femoral neck osteotomy in the DAA, including the innominate tubercle and the intertrochanteric line (ITL), which serve as the attachment site for the iliofemoral ligament [6, 7]. The presence of an ITL is universal in middle and older age groups [8]. Over the course of aging, the tendency for an individual to exhibit a more pronounced ITL appears to increase.

While these structures are consistently visible through the anterior approach [9], their relationship with the optimal level of neck resection remains undefined. An improper osteotomy height can lead to complications such as stem malalignment, leg length discrepancy, or inadequate offset restoration, all of which impact functional outcomes and implant longevity [4, 6, 10].

Given these challenges, this study aimed to investigate the anatomical relationship between the ITL and the level of femoral neck osteotomy in patients with DAA-THA. By establishing this correlation, we aim to provide surgeons with a reliable intraoperative guide, potentially reducing technical errors and enhancing postoperative outcomes. Understanding these landmarks could standardize the DAA technique, mitigating some of the difficulties associated with its learning curve.

## Materials and methods

### Study design

A prospective morphometric and cadaveric study was approved by the ethics committee of the university. This study investigated (1) the 3D anatomical relationship between the ITL and osteotomy level in CT-reconstructed femurs and (2) the accuracy of ITL-based osteotomy.

#### Part 1: 3D CT morphometric analysis

We retrospectively analyzed lower extremity CT angiography scans from 100 patients treated at our institution. A power analysis based on a pilot study (α = 0.05, power = 80%, and SD = 0.4) revealed that 58 samples were required to detect a 0.3-unit difference in key parameters. After excluding patients aged < 18 years or with hip pathology, prior fractures, or surgery, we enrolled 60 age-matched participants (30 males: 36 ± 6 years; 30 females: 34 ± 6 years; *P* = 0.16), with an overall age range of 21–46 years (mean 36 ± 7 years).

All CT data were collected from the same CT machine (Siemens AG, Erlangen, Germany) with the same scanning parameters (120 kV; 210 mA; collimation, 4 mm; table speed, 3–5 mm/s; number of slices, 80–100; and slice thickness, 1.2 mm). The 3D models of the femur were reconstructed via threshold segmentation and the interactive editing method in Amira software (FEI, USA), and a standardized coordinate system for each femoral model was constructed via the method described by Su et al. [11].

**Fig. 2:**
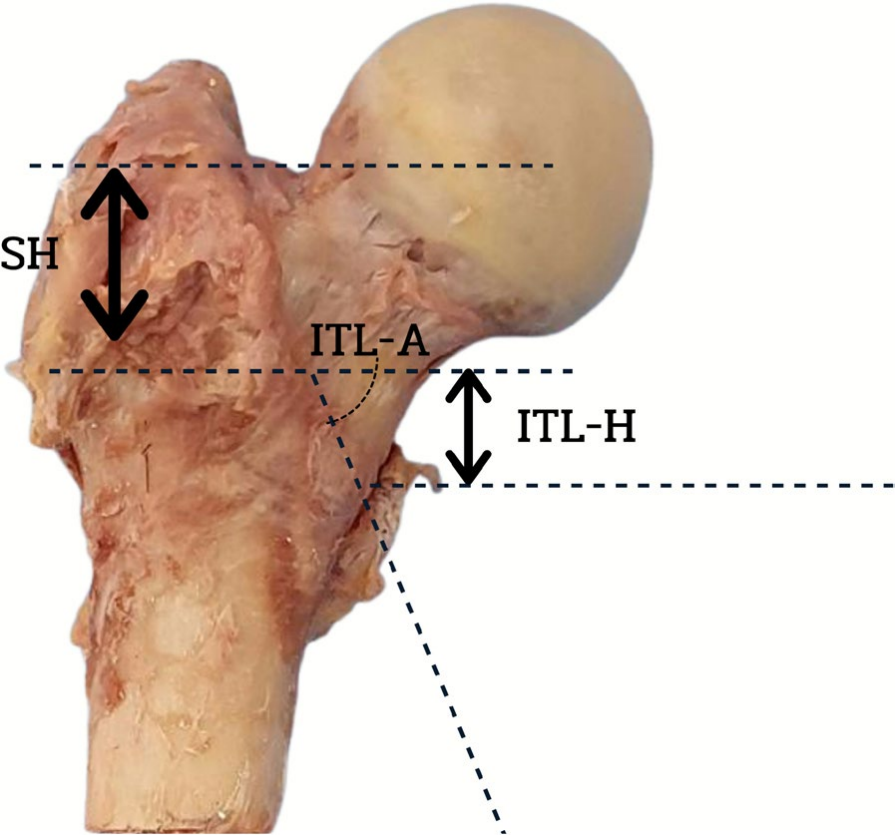
Three references used for femoral neck osteotomy: the height of an intertrochanteric line (ITL-H), the angle between the inferior ITL and level of femoral neck osteotomy (ITL-A), and the height from saddle to osteotomy level (SH)

The ITL was consistently identifiable in all 3D reconstructions. We established a standardized osteotomy plane in the coronal orientation, positioned 10 mm superior to the LT as the surgical target. Three primary parameters were quantified in Fig. 2: (1) ITL-H, the linear distance from the medial border of the inferior ITL to the planned osteotomy site; (2) ITL-A, the angular relationship between the inferior ITL and the osteotomy plane; and (3) SH, the vertical distance from the femoral saddle height to the osteotomy level. For comprehensive morphological analysis, we additionally measured femoral height (FH), defined as the longitudinal dimension from the greater trochanter apex to the intercondylar notch midpoint, and the neck‒shaft angle (NSA), calculated between the femoral head‒neck axis and the diaphyseal longitudinal axis. All measurements were performed via CAD software (SolidWorks) following 3D model standardization.

#### Part 2: Verifying validation

**Fig. 3:**
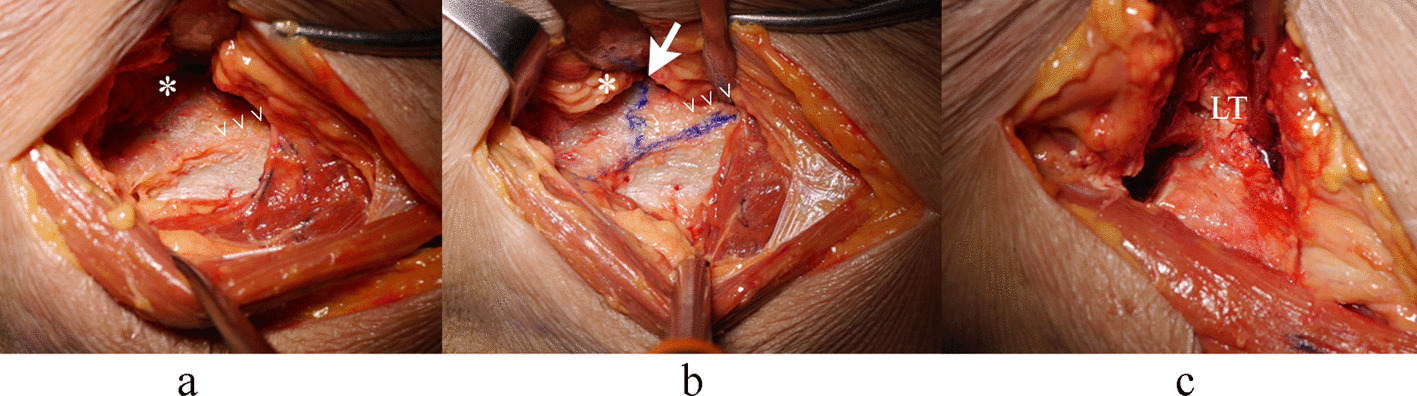
Femoral neck osteotomy level (**→**) using an inferior intertrochanteric line (ITL) (>). *illustrates the femoral neck, and LT abbreviates for lessor trochanter, which is visualized in hip external rotation after the osteotomy

Based on a priori power analysis (α = 0.05, power = 80%), twenty fresh-frozen cadaveric hips (mean donor age 73 ± 12 years; range 52–85 years) were selected for surgical validation. Specimens were excluded if they presented evidence of prior hip surgery or significant degenerative changes. All procedures were performed via the DAA with the cadaver positioned supine on a standard table. Following surgical exposure, the ITL and SH were identified and marked under direct visualization. Using the parameters derived from our CT morphometric analysis (ITL-H, ITL-A, and SH), we planned a femoral neck osteotomy 10–15 mm superior to the lesser trochanter (LT). Precise osteotomy placement was verified via intraoperative caliper measurements (Fig. 3), with the vertical distance from the osteotomy plane to the LT recorded for each sample. This experimental setup allowed quantitative assessment of the accuracy and reproducibility of our proposed anatomical landmarks in a surgical environment.

### Statistical analysis

All continuous variables are expressed as the means ± standard deviations, whereas categorical variables are reported as counts and percentages. The normality of the distributions of all measurable parameters was confirmed via the Kolmogorov‒Smirnov test. Interobserver and intraobserver reliability were assessed via intraclass correlation coefficients (ICCs), with values interpreted as follows: < 0.40, poor; 0.40–0.59, fair; 0.60–0.74, good; and ≥ 0.75, excellent. Pearson correlation analysis was used to evaluate the relationships between anatomical parameters and age, FH, and NSA. Between-group comparisons were performed via Mann‒Whitney t tests. All the statistical tests were two-tailed, with *P* < 0.05 considered statistically significant. Statistical analyses were conducted via SPSS version 26.0 (IBM Corp., Armonk, NY, USA).

## Results

Analysis of 60 3D-CT models (30 male, 30 female) revealed no significant sex differences in age (36 ± 6 vs 34 ± 6 years, *P* = 0.16) or neck-shaft angle (133° ± 2.9° vs 133° ± 2.6°, *P* = 0.93), although males presented greater femoral height (41.6 ± 2.2 cm vs 37.6 ± 1.9 cm, *P* = 0.001).

**Fig. 4:**
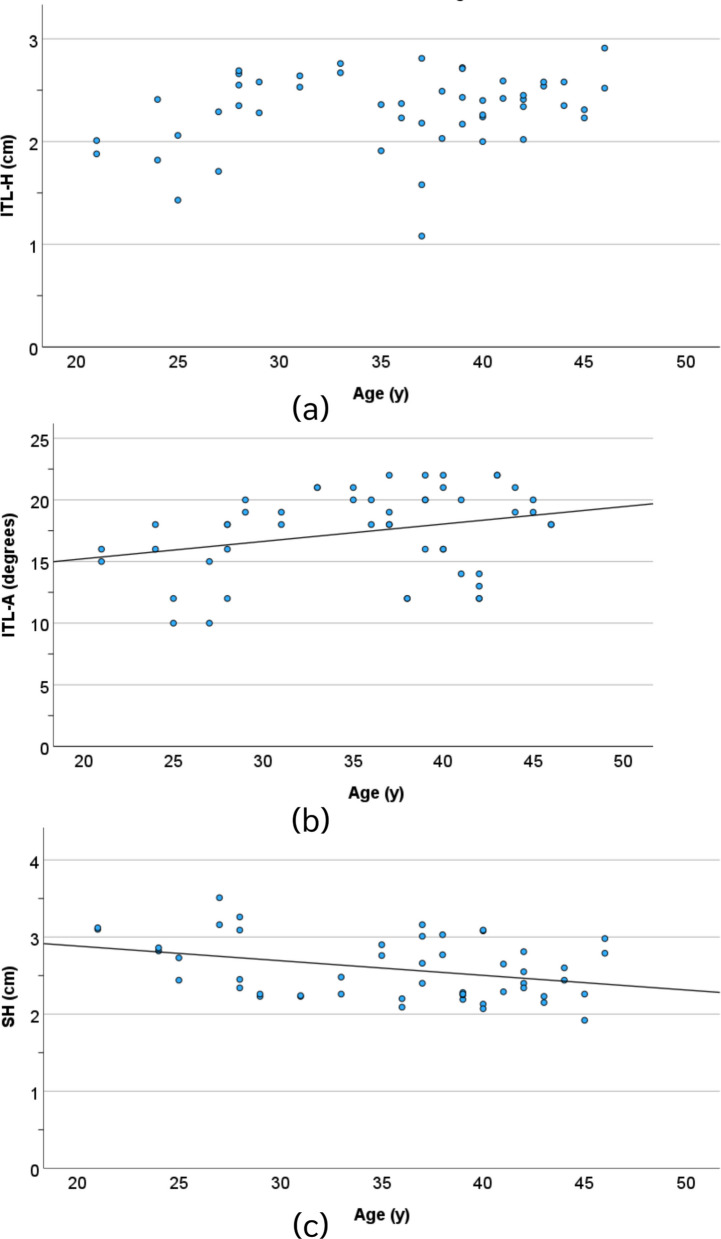
The correlation between the ITL-H (**a**), ITL-A (**b**), and SH (**c**) parameters and age

**Fig. 5:**
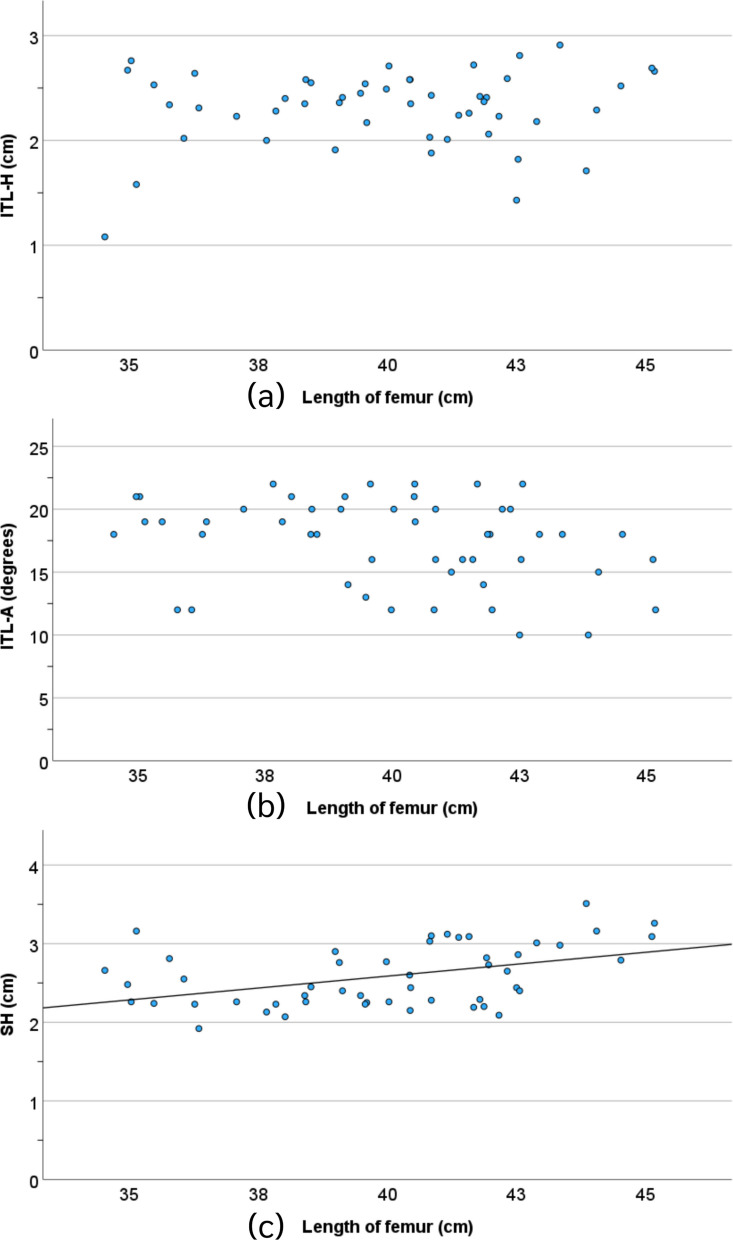
The correlation between the ITL-H (**a**), ITL-A (**b**), and SH (**c**) parameters and the length of the femur

The value of ITL-H was not significantly associated with age or femoral length (*P* = 0.06 and 0.31, respectively). In contrast, the value of ITL-A was significantly associated with age (*P* = 0.04), and the value of SH was significantly associated with both age and femoral length (*P* = 0.01 and 0.001, respectively), Figs. 4 and 5.

Interobserver reliability was excellent for the ITL-H (ICC = 0.86) and SH (0.88) but poor for the ITL-A (0.25). The mean values of ITL-H, ITL-A, and SH were 23 ± 4 mm, 17.4° ± 3.5°, and 26 ± 4 mm, respectively, to achieve a cutoff level of 10 mm above the LT via the bony landmarks used to visualize the DAA, as shown in Table 1**.** While ITL-H showed no sex difference (23 ± 3.4 mm vs 23.1 ± 4.1 mm, *P* = 0.96), significant differences existed for ITL-A (15.8° ± 3.4° vs 19.9° ± 1.4°, *P* = 0.001) and SH (27.2 ± 3.9 mm vs 23.9 ± 3 mm, *P* = 0.002). ITL-H was not correlated with age (*P* = 0.063), femoral height (*P* = 0.31), or NSA (*P* = 0.41).

**Table 1:** Relationships between bony landmarks and the level of femoral neck osteotomy measured 10 cm above the lesser trochanter

	**Mean total**	**Male (mean ± SD)**	**Female (mean ± SD)**	***P* value (Mann‒Whitney t test)**
ITL-H (mm)	23 ± 4	23.1 ± 3.4	23.1 ± 4.1	0.96 (− 0.219–0.208)
ITL-A (degree)	17.4 ± 3.5	15.8 ± 3.4	19.9 ± 1.4	0.001 (− 5.76– − 2.5)
SH (mm)	26 ± 4	27.2 ± 3.9	23.9 ± 3	0.002 (0.12–0.53)
FH (cm)	40 ± 2.8	41.6 ± 2.2	37.6 ± 1.9	0.001 (2.71–5.15)
NSA (degree)	133 ± 2.8	133 ± 2.9	133 ± 2.6	0.93 (− 1.71–1.57)

The ITL is composed of converging medial and lateral arms and does not form a single linear structure. As a result, defining a consistent reference line for angular measurement between the ITL and the osteotomy plane is subject to observer interpretation, leading to variability and low interobserver agreement. Given this limitation, ITL-A was not considered a reliable parameter for intraoperative guidance and was therefore excluded from surgical validation. Owing to their excellent interobserver reliability (ICC = 0.86 for ITL-H, 0.88 for SH), these two parameters were selected for surgical validation.

With respect to ITL-H, the inferior portion was consistently defined as the most distal footprint of the medial arm of the intertrochanteric line. This region forms a discrete bony prominence on the anterior aspect of the proximal femur and was identifiable in all patients included in the study. Using predetermined reference values (ITL-H: 23 mm; SH: 26 mm) to guide osteotomy placement, quantitative analysis revealed significantly greater precision with ITL-H guidance (%RSD 21.8 vs 54.5, *P* < 0.01). The ITL-H method produced a median resection level of 12.5 mm (IQR 10.2–14.1 mm) above the lesser trochanter, whereas it was 7.1 mm (IQR 5.3–9.8 mm) with SH guidance.

Clinically, the use of the ITL-H as a reference demonstrated an 80% success rate in achieving the target 10–15 mm (12.6 ± 0.7 mm) osteotomy zone, with all patients (100%) maintaining the minimum required 5 mm (7 ± 1.7 mm) of calcar bone stock. These findings confirm that ITL-H is a more reliable intraoperative landmark for consistent femoral neck resection in DAA-THA patients.

## Discussion

**Fig. 6:**
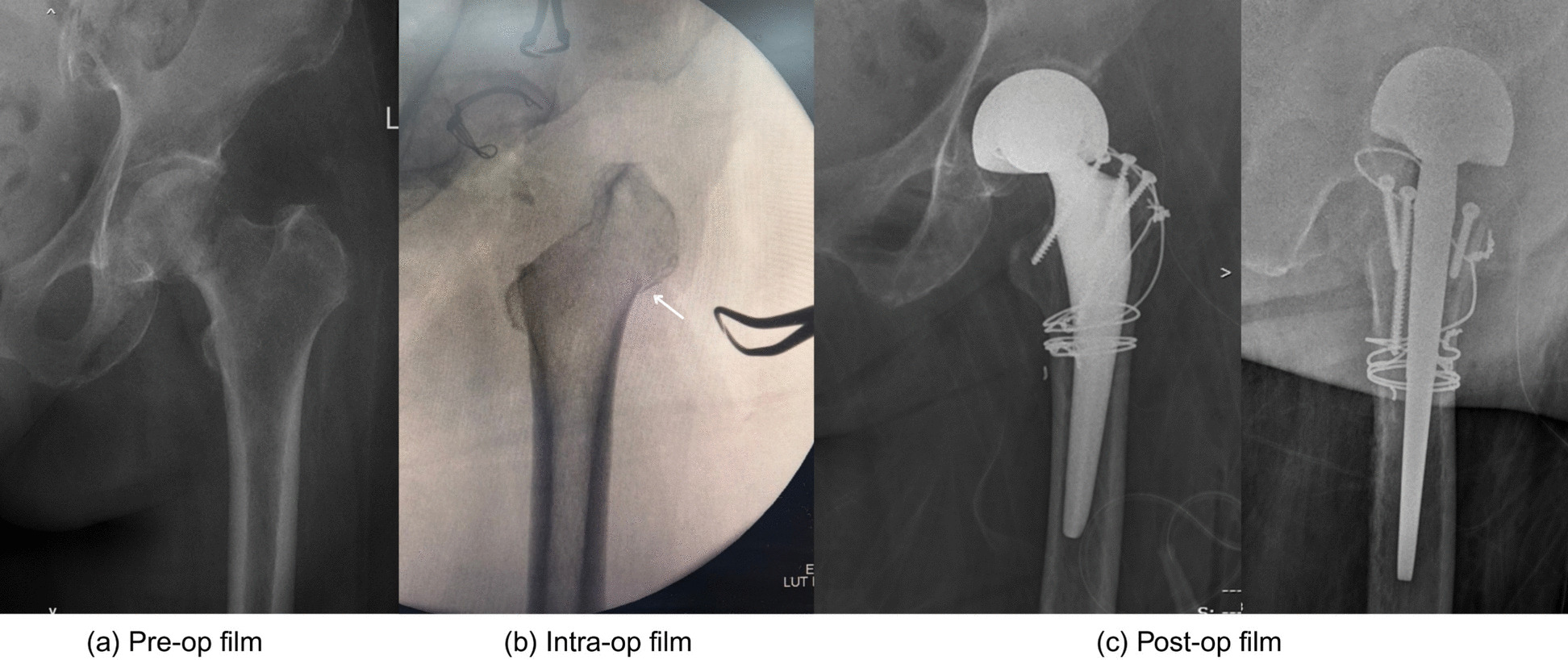
Excessive calcar resection and a greater trochanteric fracture (arrow) resulting from an inaccurate femoral neck osteotomy during DAA bipolar hemiarthroplasty for a femoral neck fracture

Inaccurate femoral neck osteotomy is a well-recognized technical challenge in DAA-THA and remains an important contributor to early technical errors, particularly during the learning curve of the approach [2, 3, 5], as Fig. 6**.** Most steps would require a high level of technical demand and may involve multiple intraoperative fluoroscopic checks. In contrast to posterior approaches, in which the LT and calcar are easily identified, the DAA lacks a universally accepted bony landmark for guiding an in-situ femoral neck osteotomy. Consequently, many surgeons rely on experience-based estimation and fluoroscopic estimation, both of which may increase variability in osteotomy height and orientation. Previous studies have demonstrated that such variability can adversely affect leg length restoration, femoral offset, stem alignment, and overall biomechanical reconstruction following THA [6, 10].

The clinical consequences of an inaccurate femoral neck osteotomy are especially relevant in patients with pre-existing leg length discrepancy or lower limb asymmetry, conditions that are not uncommon among individuals undergoing THA. Even small deviations in osteotomy height may translate into clinically meaningful postoperative imbalance, patient dissatisfaction, or the need for intraoperative correction [10]. Excessive resection may compromise calcar bone stock and increase the risk of stem subsidence, whereas insufficient resection may impede stem seating or necessitate repeated osteotomies, thereby increasing operative time and fracture risk [6]. These considerations underscore the need for a reproducible intraoperative reference that can reduce variability while remaining practical for routine clinical use.

The present study demonstrates that the ITL-H provides a consistent and reliable anatomical reference for guiding femoral neck osteotomy during DAA-THA. Using three-dimensional CT-based morphometric analysis, we identified a stable spatial relationship between the most distal portion of the ITL and the LT. This relationship was independent of sex, age, femoral height, and neck–shaft angle, suggesting that ITL-H is robust across a wide range of normal anatomical variations. Importantly, cadaveric validation confirmed that an osteotomy performed approximately 23 mm above the ITL reliably achieved a target resection of 10–15 mm above the LT while preserving adequate calcar bone stock.

Previous anatomical studies have described potential landmarks relevant to the anterior approach, such as the innominate tubercle or the insertional footprints of the iliofemoral ligament, and have demonstrated their anatomical consistency [9, 12]. However, these investigations were largely descriptive and did not define a quantitative osteotomy level relative to the LT, nor did they assess intraoperative applicability [13, 14]. In contrast, the present study extends prior work by combining three-dimensional anatomical analysis with cadaveric surgical validation, thereby providing a clinically actionable reference that can be directly translated into intraoperative practice during DAA-THA.

Individualized preoperative planning remains essential for accurate biomechanical restoration in THA. Nevertheless, planning femoral neck osteotomy based solely on plain radiographs has inherent limitations related to magnification error, pelvic tilt, and rotational malposition, which obscure the true three-dimensional relationship between the ITL, the LT, and the femoral neck axis [10]. While CT-based planning offers greater anatomical accuracy, routine use is constrained by cost, radiation exposure, and availability. In this context, ITL-H should be regarded not as a replacement for preoperative templating but as a reliable intraoperative adjunct that helps bridge the gap between planning and execution when advanced imaging is unavailable.

Technological advances such as computer navigation, robotic-assisted surgery, and patient-specific instrumentation may further improve the accuracy of femoral neck osteotomy by enabling real-time assessment of leg length, offset, and resection planes [6]. However, these technologies are not universally accessible and may increase operative cost and workflow complexity. Accordingly, a reproducible anatomical landmark such as ITL-H remains clinically relevant in routine DAA-THA practice and may also serve as an intraoperative validation point when advanced technologies are used.

Several limitations of this study should be acknowledged. The ITL should be used with caution as an anatomical reference in younger patients (< 30 years of age), as it may be absent in approximately 14% of individuals in this age group [8]. The anatomical analysis was performed in hips without significant deformity; therefore, extrapolation to conditions such as developmental dysplasia of the hip or post-traumatic deformity should be undertaken with caution. In addition, CT-based modeling may not fully replicate intraoperative soft-tissue constraints, and clinical outcome data were not assessed. Despite these limitations, the consistency of ITL-H across demographic variables and its successful cadaveric validation support its applicability as a practical intraoperative reference. Future studies should evaluate its performance in complex hip pathology and examine the relationship between osteotomy accuracy and postoperative clinical outcomes.

## Conclusion

Inaccurate femoral neck osteotomy remains a technical challenge in DAA-THA due to limited femoral exposure, altered femoral orientation, and the absence of a standardized intraoperative landmark [2, 3, 5, 6]. This study demonstrates that ITL-H serves as a practical intraoperative adjunct that complements conventional templating and may help reduce variability in femoral neck osteotomy during routine DAA-THA. An osteotomy level approximately 23 mm above the ITL-H defines a safe lower margin to avoid excessive calcar bone resection rather than a fixed resection target. Accordingly, individualized preliminary osteotomy based on preoperative templating remains essential, with intraoperative adjustment according to patient-specific ITL-H to achieve accurate biomechanical restoration.

## Data Availability

No datasets were generated or analysed during the current study.
